# An analysis of child suicide from three centers (2008–2017)

**DOI:** 10.1007/s12024-022-00505-1

**Published:** 2022-07-25

**Authors:** Kelly L. Olds, Rexson Tse, Simon Stables, Andrew M. Baker, Kathryn Hird, Neil E. I. Langlois, Roger W. Byard

**Affiliations:** 1 School of Medicine, The University of Notre Dame Australia, Fremantle, Australia; 2 LabPLUS, Auckland City Hospital, Auckland, New Zealand; 3 Hennepin County Medical Examiner, Minneapolis, USA; 4 School of Biomedicine, The University of Adelaide, Adelaide, Australia; 5 Forensic Science South Australia, Adelaide, Australia

**Keywords:** Child, Youth suicide, Hanging, Gunshot, Australia, New Zealand, United States

## Abstract

Although the overall suicide rate worldwide has changed minimally over the past 100 years, different trends have been observed over time in the USA, Australia, and New Zealand (NZ). However, few studies have focused on suicides in children (< 18 years), making evaluation of possible trends difficult. The last 20 years has also seen an increase in childhood obesity, eating disorders, and body image issues for children in many developed nations; however, few studies have shown whether a significant proportion of child suicides have an abnormal BMI. The current study evaluates child suicides (from 2008 to 2017) in South Australia (Australia), compared with the jurisdictions of Auckland (NZ) and Hennepin County (USA). Demographic data (age, sex, ethnicity), body mass index (BMI), the number of cases of youth suicide, and the method of suicide from these three regions were collected and analyzed. Across the 10-year period, the jurisdiction of Auckland had a downward trend, while Hennepin County and South Australia had increasing numbers of cases. The most common method of child suicide in all centers was hanging, occurring in > 80% of cases in South Australia and Auckland and 56% in Hennepin County. Hennepin County had a greater proportion of suicides using firearms (28%), compared to 1.9% in Auckland and 5.1% in South Australia. Unusual means of suicide were used less frequently by youth than previously.

## Introduction

The overall suicide rate worldwide has been found to have changed little over the past 100 years [1] although different trends have been observed in particular countries. Both the USA and Australia showed an initial increase in the suicide rate for young adults (aged 15–24 years) from the 1970s until the late 1990s, whereas in the early 2000s, there was a significant decrease [2–4]. This is in contrast to New Zealand, which had an increase in the suicide rate within the same age group [5]. However, few studies to date have focused on the suicide rate of children; consequently, there is limited prior knowledge from which to determine trends. In studies of suicide rates in children, the rates in the USA and Australia appeared to be static [6, 7]. Conversely, in New Zealand, the suicide rate of children was found to be increasing [8]. It has been noted that while suicide rarely occurs in childhood, there is a dramatic increase in cases in late adolescence [9].

A study performed by Byard and colleagues (2000) on youth and adolescent (under 17 years of age) suicide in Adelaide (South Australia, Australia) and San Diego (CA, USA) demonstrated that the preferred methods of suicide used by youth differed to those employed in older age groups [7]. While hanging was still found to be a common method of child suicide, there was an increased percentage of less common methods in those under 18 years [7]. These methods included jumping from buildings or in front of transport such as trains [7]. Furthermore, this study demonstrated a difference in the frequencies of certain methods of suicide between the two centers, with firearms used preferentially in the San Diego cohort, in contrast to South Australia [7]. Given that this study was performed almost two decades ago, it is considered that trends in child suicides may have changed in the intervening period.

Over the last two decades, there has been an increasing proportion of children and adolescents classified as overweight or obese. Both negative body image and having a BMI classified as underweight or obese/morbidly obese have been shown to increase suicidal ideation in adolescents [10, 11]. There are, however, no available studies that the authors could find that attempt to determine whether there are a significant proportion of child and adolescent suicide cases with high or low BMIs.

The current study thus provides updated information on child suicides in Australian (Adelaide, South Australia), US (Minneapolis, Hennepin County), and New Zealand (Auckland) centers.

## Materials and methods

Cases of suicide in those under the age of 18 years from 2008 to 2017 within jurisdictions of South Australia (Australia), Hennepin County (USA), and Auckland (New Zealand) were searched via departmental autopsy databases. All cases within the study timeframe and age range were included. Ethics approval was obtained from each jurisdiction.

Once cases were identified, data were then collected from autopsy electronic records and/or archived paper autopsy reports. Demographic variables that were collected included age, sex, BMI, and ethnicity. No identifiable information was recorded. Outcome variables that were collected included method of suicide, blood alcohol concentration, presence of illicit drugs (including cannabis), and psychiatric medications.

In order to calculate the suicide rate (per 100,000 young persons) and determine the trends for each center, estimate resident population data were obtained from the relevant government body in the USA, New Zealand, and Australia [12–14]. The population data for each center were available in 5-year age groupings (0–4, 4–9, 10–14, and 15–19 years of age) for each year within the period of 2008–2017. These data were collected and used to scale the number of suicides into a per 100,000 persons rate, to determine whether the incidence of suicides in each center had changed over time, while accounting for the population size. Similarly, population data for each country (USA, New Zealand, and Australia), as well as the number of child suicides in each nation, were collected for each year in the period of 2008–2017, and a national suicide rate was calculated to determine whether the selected centers in this study were representative of their country. Due to differences in data presentation of child suicides between the nations, the USA and New Zealand show the national suicide rate per 100,000 young persons 19 years and under, whereas Australia shows the national rate for young persons 17 and under.

### Analysis

Statistical analysis was performed using Python, with results presented in the form of descriptive statistics (frequencies and proportions) and ordinary least squares linear regression to determine if there was a significant change in the suicide rate from that previously observed in American and Australian states. The suicide rate per 100,000 young persons was then calculated using the number of child suicides (under 18 years) within a given year, divided by the estimate resident population data of those 0–19 years within that time period for each jurisdiction. The model: number of suicides = beta0 + beta1 * *T* + error was then run, with *T* as the time trend, and tested to see if beta1 was significantly different from zero by using a *t*-test. Significance was set at 1%.

## Results

### Hennepin County, Minneapolis, USA

**Fig. 1:**
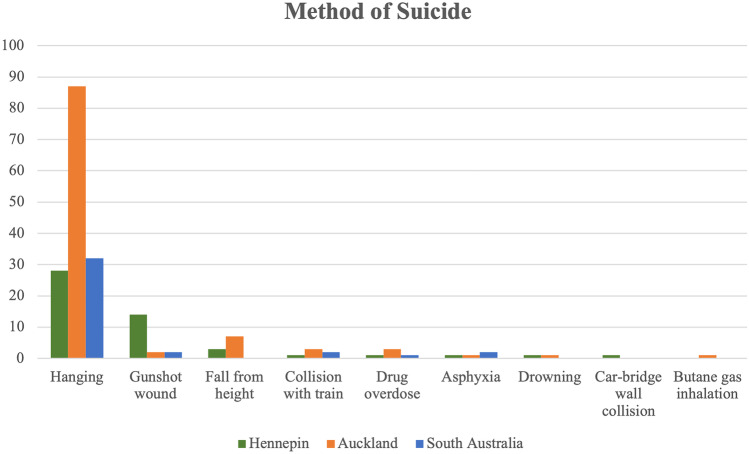
Cases of child suicide in Hennepin County, South Australia, and Auckland from 2008 to 2017

**Fig. 2:**
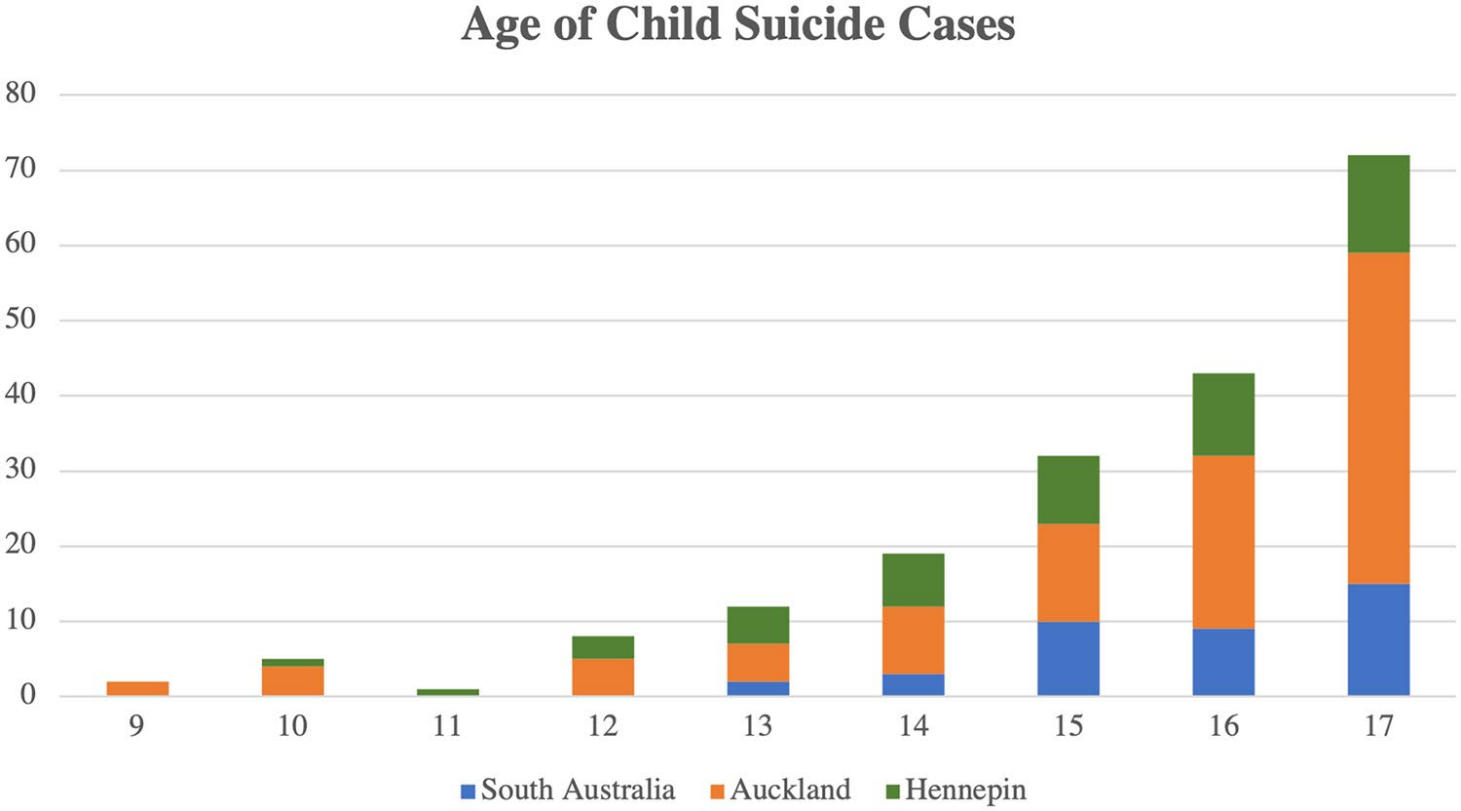
Cases of child suicide per month in the jurisdictions of Hennepin County, South Australia, and Auckland from 2008 to 2017

**Fig. 3:**
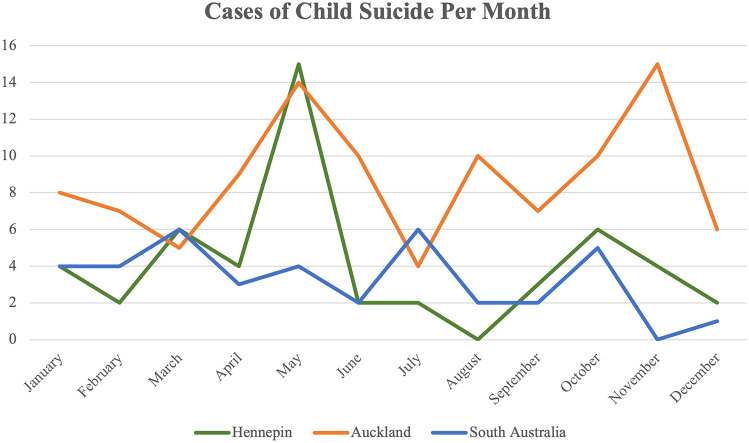
Age of child suicide cases in South Australia, Hennepin County, and the Auckland region across a 10-year period (2008 to 2017)

From 2008 to 2017, there were 50 child suicides (3.6% of 1404 total suicides). The number of child suicides each year ranged from three to 11 cases, with 11 occurring as a peak in 2014 (Fig. 1). Between 2008 and 2017, cases of child suicide occurred in certain months with wide discrepancy, ranging from two to 15 times. The month in which most youth suicides occurred was May, with 15 cases during the 10-year period (Fig. 2). The age range for child suicides was from 10 to 17 years (median 15), with most cases occurring in the 17-year age group (Fig. 3). There was a male predominance, comprising two-thirds of cases (33 cases, 66%), compared to females (17 cases, 34%). In six cases (12%), the decedent was classed as overweight, and in three cases (6%), the decedent was obese. There were two cases (4%) classified as underweight. The majority of the decedents were within the normal BMI range (78%). The majority of decedents in this population were Caucasian (37, 74%), with the remaining thirteen cases being African-American (18%), Hispanic (2%), Native American (4%), and Asian (2%).

**Fig. 4:**
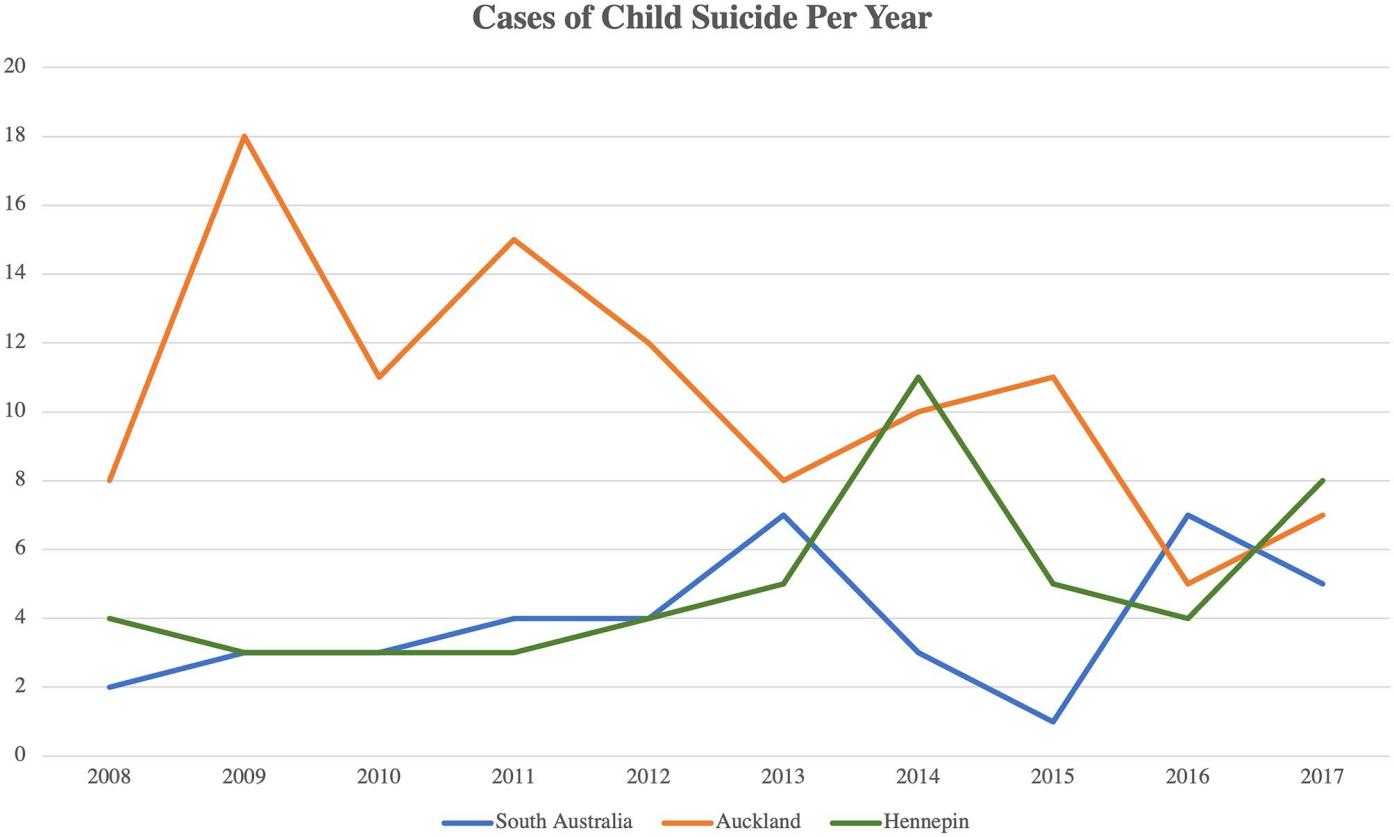
Frequency of child suicide methods in the jurisdictions of Hennepin County, South Australia, and Auckland between 2008 and 2017

The most commonly selected method of suicide was hanging, occurring in 56% of cases (Table 1, Fig. 4). Gunshot wounds to the head were the next most common method, accounting for 28% or 14 cases (Table 1, Fig. 4). Death due to a fall from height comprised 6% of cases (Table 1, Fig. 4). There were singular cases of collision with a train, collision with a bridge wall (decedent in a vehicle), drowning, suffocation, and drug overdose (Table 1, Fig. 4). Alcohol was reported in seven (14%) out of 50 cases, with blood levels ranging from 0.009 to 0.33% (Table 2). Illicit drugs were detected in three out of 50 cases (6%) (Table 2). All 50 cases were assessed for the presence of psychiatric medications which were detected in 13 cases (26%) and included antidepressant (11 cases, 22%), anxiolytic (5 cases, 10%), and antipsychotic (1 case, 2%) classes of medications (Table 2).

**Table 1:** Method of suicide for cases in Hennepin County, South Australia, and Auckland from 2008 to 2017

**Method of suicide**			
	**Hennepin**	**Auckland**	**South Australia**
**Hanging**	28 (56%)	87 (82.91%)	32 (82.1%)
**Gunshot wound**	14 (28%)	2 (1.9%)	2 (5.2%)
**Fall from height**	3 (6%)	7 (6.7%)	0 (0%)
**Collision with train**	1 (2%)	3 (2.9%)	2 (5.2%)
**Drug overdose**	1 (2%)	3 (2.9%)	1 (2.6%)
**Asphyxia**	1 (2%)	1 (1%)	2 (5.2%)
**Drowning**	1 (2%)	1 (1%)	0 (0%)
**Car-bridge wall collision**	1 (2%)	0 (0%)	0 (0%)
**Butane gas inhalation**	0 (0%)	1 (1%)	0 (0%)

**Table 2:** Toxicology findings in child suicide cases from 2008 to 2017 for the jurisdictions of Hennepin County, South Australia, and Auckland

**Toxicology**			
	**Hennepin**	**Auckland**	**South Australia**
**Illicit drugs**			
**None**	47 (94%)	88 (84%)	36 (92.3%)
**Cannabis**	0 (0%)	0 (0%)	10 (25.6%)
**Amphetamines**	2 (4%)	0 (0%)	2 (5.2%)
**Cocaine**	1 (2%)	0 (0%)	0 (0%)
**Cathinone**	0 (0%)	1 (1%)	0 (0%)
**LSD**	0 (0%)	0 (0%)	1 (2.6%)
**Not submitted**	0 (0%)	16 (15.2%)	0 (0%)
**Alcohol**			
**Present**	7 (14%)	26 (24.8%)	10 (25.6%)
**Absent**	43 (86%)	63 (60%)	29 (74.4%)
**Not submitted**	0 (0%)	16 (15.2%)	0 (0%)
**Psychiatric drugs**			
**None**	37 (74%)	83 (79%)	32 (82%)
**Anti-depressants**	11 (22%)	4 (3.8%)	4 (10.3%)
**Anti-psychotics**	1 (2%)	3 (2.9%)	1 (2.6%)
**Anxiolytics**	5 (10%)	2 (1.9%)	2 (5.1%)
**Anti-ADHD**	0 (0%)	0 (0%)	0 (0%)
**Not submitted**	0 (0%)	16 (15.2%)	0 (0%)

### Jurisdiction of Auckland, New Zealand

Between 2008 and 2017, there were 105 cases of child suicide, which comprised 6.4% of 1638 total suicides. The age range for child suicides was from 9 to 17 years of age (median 16) (Fig. 3). Slightly more than half of these cases were male (54 cases, 51.4%) and slightly less than half were female (51 cases, 48.6%). The number of suicides each year ranged between five and 18, with 18 suicides occurring in 2009 (Fig. 1). May and November were the two months in which most child suicides occurred (Fig. 2). There were 14 cases that occurred in May and 15 in November, compared with only four cases in July from 2008 to 2017 (Fig. 2). Two-thirds (70 cases, 66.6%) of the cases were within the normal BMI range. In ten cases (9.5%), the decedents fell into the overweight BMI range, and in 25 cases (23.8%), they were deemed obese. Maori children and adolescents comprised 59% (62 of 105 cases) of the study population. The ethnicities listed in the remaining cases were Caucasian (25 cases, 23.8%), Pacific Islander (five cases, 4.8%), Indian (four cases, 3.8%), Samoan (one case, 1%), and Asian (one case, 1%). In two cases (1.9%), ethnicity was not listed.

The method of suicide selected in the Auckland group was mainly hanging, with 87 cases (82.9%) (Table 1, Fig. 4). Other methods of suicide chosen were fall from height (seven cases, 6.7%), collision with train (three cases, 2.9%), drug overdose (three cases, 2.9%), gunshot with 0.22 caliber rifle (two cases, 1.9%), plastic bag asphyxia (one case, 1%), drowning (one case, 1%), and butane gas inhalation (one case, 1%) (Table 1, Fig. 4).

In 16 of the 105 cases, toxicology was not undertaken (15.2%) (Table 2). Alcohol was not detected in 63 of the 89 cases sent for toxicological analysis (60%). In the 40% of cases that did have a blood alcohol concentration reported, concentrations ranged between 0.009 and 0.236%. Illicit drugs were detected in 13 of the 89 cases (14.6%) where toxicology was requested (Table 2). In 12 cases, the illicit substance was cannabis; the other case was cathinone (Table 2). In most cases (83 cases, 79%), there were no psychiatric medications detected via post-mortem toxicology (Table 2). Drug classes that were detected included antidepressants (4 cases, 3.8%), anxiolytics (2 cases, 1.9%), and anti-psychotics (3 cases, 2.9%) (Table 2).

### South Australia, Australia

There were a total of 39 cases of child and adolescent suicide from 2008 to 2017 (2.4% of 1661 total suicides). The number of cases that occurred in any given year ranged from one to seven (Fig. 1). The number of cases of suicide occurring in a particular month ranged between one and six (Fig. 2). March, July, and October were found to have the highest number of cases with six, six, and five cases, respectively (Fig. 2). The age range in South Australia cases was from 13 to 17 years, with a median of 16 years (Fig. 3). The highest number of cases occurred in the 17-year-old age group (Fig. 3). Slightly more than one-third were females (14 cases, 35.9%), with 25 of the 39 cases being males (64.1%). Greater than three-quarters of decedents were within the normal BMI range (30 cases, 76.9%). Six cases (15.4%) were classified as overweight and three (7.7%) were deemed underweight. The majority of cases were Caucasian (36, 92.3%), with single cases involving Aboriginal (2.6%), African (2.6%), and Asian (2.6%) decedents.

The method of suicide employed predominantly was hanging, with 32 cases (82.1%) (Table 1, Fig. 4). Gunshot wounds with 0.22 caliber rifles, plastic bag asphyxia, and cases where the decedent died via collision with a train were the other methods of suicide in this study population, each with two cases (5.1%) (Table 1, Fig. 4). There was one case involving MDMA toxicity (2.6%) (Table 1, Fig. 4). In 29 of the 39 cases, no alcohol was present (74.4%) (Table 2). Blood alcohol concentrations ranged between 0.016 and 0.164%. In terms of illicit drugs, cannabis was the most commonly detected drug in 10 of the 39 cases (25.6%) (Table 2), with values between 2 and 37 µg/L present. MDMA, LSD, and methylamphetamine were detected in single cases (Table 2). In only one of these three cases was there combined intoxication with cannabis and another illicit drug. In 27 of the 39 cases (69.2%), there were no illicit drugs detected. Psychiatric medications were detected in preserved peripheral blood in seven cases (17.9%) and included antidepressant (4 cases, 10.3%), anxiolytic (2 cases, 5.2%), and antipsychotic (1 case, 2.6%) classes of medications (Table 2).

### Suicide rate

**Fig. 5:**
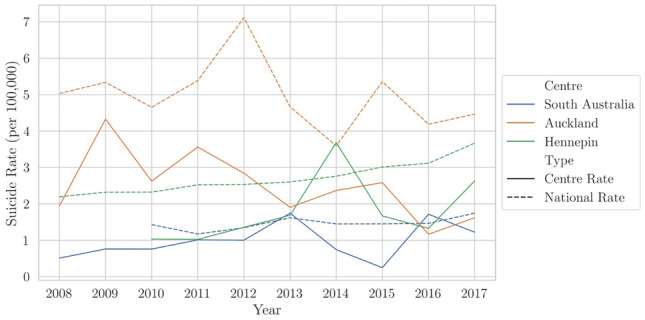
The suicide rate per 100,000 young persons (19 years and under) per year in South Australia, Hennepin County, and Auckland from 2008 to 2017 (solid colored lines for each center). The trend is downward for Auckland and upward for South Australia and Hennepin County. The dashed colored lines represent the national suicide rate (per 100,000 young persons 19 years and under for the USA and New Zealand, and 17 years and under for Australia, due to differences in Australia’s presentation of child suicide data). The suicide rate in each center appears to follow the same trend as its respective nation, just at a different level

Using the linear regression model to plot the suicide rate in each jurisdiction showed that the suicide rate was trending downward in the jurisdiction of Auckland, whereas Hennepin County and South Australia were trending upward during the period of 2008–2017 (Fig. 5). The coefficients were 0.064 for South Australia (trend of 1 additional death every 15.6 years); 0.186 for Hennepin County (1 additional death every 5.4 years); and − 0.180 for the jurisdiction of Auckland (1 fewer death every 5.6 years). All three trends were statistically significant (*p* < 0.01). When comparing the results of these centers with their respective national suicide rates, the centers appear to follow the same trend as their nation overall, merely at different levels (Fig. 5).

## Discussion

The population of Hennepin County, Minneapolis, was approximately 1.2 million in 2017 [12], with 304,043 persons 19 years or under [15, 16]. The population of South Australia was approximately 1.7 million, with 409,395 persons aged 19 or younger in 2017 [14]. The population of the Auckland region was approximately 1.6 million in 2017, with 432,920 persons under 19 years of age [13].

New Zealand has previously had more child and adolescent suicides than other developed nations, including Australia and the USA [8]. Even though the jurisdiction of Auckland had a larger total number of suicides per year and the highest proportion of child suicides (105 child suicides, 6.4% of 1638 total suicides) in our study when compared with South Australia (39 child suicides, 2.4% of 1661 suicides) and Hennepin County (50 child suicides, 3.6% of 1404 total), the jurisdiction of Auckland’s rate has been trending downward, whereas both South Australia and Hennepin county’s rate was trending upward during the period of 2008–2017.

There was a similar age distribution observed for each of the jurisdictions, with most suicides occurring between 15 and 17 years. Children tend to have an understanding of suicide by 8 years of age [17]. The youngest age of suicide reported among the three jurisdictions was 9 years. Analysis of child suicide by month for each jurisdiction shows that child suicides are not seasonal.

In both South Australia and Hennepin County, only approximately one-third of the child suicides were female. This is in keeping with previous literature on child suicide, which has reported male suicides to be higher than females [7, 18]. A study performed in 2015 by Mendes and colleagues had similar results, with two-thirds of child suicides being males [19]. It has also been noted that females attempt suicide more frequently than males, but that males have a higher rate of completed suicide [20]. This may be attributed to males selecting more violent means of suicide from which rescue attempts are less likely to be successful, or from males being less likely to seek help when they are suicidal [20]. In Hennepin County—the center with the most variable methods of suicide—in cases that involved collisions or falls from height, and all but two of the suicides from gunshot wounds, the decedent was male. In contrast to the gender distribution seen in Hennepin and Adelaide, in the jurisdiction of Auckland, approximately half of the cases of the youth suicides were female. This is different to findings from a previous study performed in New Zealand from 1989 to 1998, where greater than two-thirds (72.1%) were male [8].

It is reported in the literature that youths at the extremes of BMI have a negative body image and are at increased risk of self-harm and suicidal ideation [10, 11]. In a study performed in 2007 that surveyed middle school youth in the USA, females who perceived themselves to be overweight were more likely to report both suicidal thoughts and actions [21]. Males were more likely to report suicidal thoughts and actions if they were overweight or underweight [21]. In our study, decedents from Hennepin County and South Australia had similar proportions of BMI, with approximately three-quarters of cases being within the normal weight range and 4% (Hennepin) and 7.7% (South Australia) falling within the underweight category. In Auckland, however, one-third of the decedents were either overweight or obese. Four of the five underweight cases in this study were males. Overall, these findings show that a number of the child suicide cases in this study were at the extremes of BMI and that BMI may be an important risk factor to consider in child suicides.

In Auckland, 59% of child suicides were of Maori ethnicity. This proportion aligns with that of an earlier study performed in New Zealand, which found that Maori children and adolescents accounted for 57.4% of youth suicides [8]. Although Maori children represented the highest proportion of child suicides in Auckland during this study period, they comprised only 4.2% of the 0–19 population in 2013 [13]. In contrast to Auckland, the majority of decedents in both South Australia and Hennepin County were Caucasian.

The most commonly employed method of suicide across all jurisdictions was hanging. There were fewer cases of drug overdose, falls from height, and collisions with vehicles/objects than have been previously reported in similar Australian, American, and New Zealand studies [5, 7]. Cases were considered suicide rather than accidental from drug overdose when there was additional evidence available such as a suicide note or a stated intention by the decedent. Alcohol and cannabis were the most commonly reported drugs/substances of abuse. Cannabis was detected more frequently than alcohol in the South Australian and Hennepin cases, whereas, in Auckland, alcohol was detected more frequently. In most cases of youth suicide, however, no illicit drugs or alcohol were detected.

In Hennepin County, the second most common method of suicide was gunshot; in contrast, cases of suicide by gunshot wound were significantly less frequent in the Adelaide and Auckland centers. These findings appear to reflect a difference in the availability and access to firearms between Hennepin and the two non-US centers. A study by Miller and Azrael in 2020 found that children and adolescents having access to firearms increases their risk of suicide, and that 40% of suicides in this age group in the USA are by gunshot wound [22].

### Limitations

A limitation of this study is that the suicide rates were determined using estimate resident population data for 0–19 years of age, when cases of suicide were only collected for those aged less than 18 years. This was unavoidable given the way in which population data are presented (in 5-year age groupings). It is acknowledged that there are an additional 2 years (18 and 19) in this age bracket for which cases of suicide are not included; however, this was applied to all jurisdictions, and therefore each jurisdiction would be expected to be affected similarly. When comparing the findings in each center with their national suicide rate across the same period, while the USA and New Zealand had both national population and suicide data available for those 19 years and under, Australia had population data for those 19 years and under but only reported child suicides for those 17 years and younger, meaning that the national suicide rate in Australia is likely an underestimate.

There are also differences in how the manner of death is determined between the jurisdictions. In Hennepin County, this is based on the opinion of the pathologist, whereas in Auckland and South Australia, it is determined by the coroner using information provided by police, family, and the forensic pathologist. This could potentially have influenced results, given that only cases with manner classified as “suicide” were included in this study.

Child suicide is an uncommon occurrence and so it is possible that cases may exist that are suicides but have been assigned as accidents—this is more likely to occur in this age group compared with those above the age of 18, due to unusual methods such as colliding with a train/other vehicle or a fall from height. However, unusual methods were less commonly seen in this study than have been seen previously in studies from these jurisdictions. Similar to the results seen in this article, a study from the UK by Zainum and Cohen in 2017 found that unusual methods of suicide were not frequently employed by children [23].

## Conclusion

Auckland had both the most cases of child suicide and the largest proportion of child suicides from 2008 to 2017 but their suicide rate was trending downward during this period. The suicide rates for Hennepin County and South Australia between 2008 and 2017 both trended upward. The most common method of suicide across all centers was hanging; this occurred in over 80% of cases in both South Australia and Auckland and 56% in Hennepin County. There were a greater proportion of suicides using firearms in Hennepin County than the other centers (28%, compared with 1.9% for Auckland and 5.1% for South Australia). This most likely arises from ready access to a means of self-destruction [24]. Unusual means of suicide, such as poisoning, collision with a structure or vehicle, and falls from height, were used less frequently by youth than previously reported.

## Key points

- From 2008 to 2017, suicides in children (< 18 years) had a significant downward trend in Auckland, New Zealand.
- In contrast, over the same period, suicides in children significantly increased in Hennepin County, USA, and in South Australia, Australia (*p* < 0.01).
- The most common method of child suicide in all centers was hanging, occurring in > 80% of cases in South Australia and Auckland and 56% in Hennepin County.
- Hennepin County had a greater proportion of suicides using firearms (28%), compared to 1.9% in Auckland and 5.1% in South Australia.
- Suicide rates for children have changed over time but in significantly different ways in different communities.

## References

[1] Goldney RD, Harrison J (1998). Suicide in the elderly: some good news. Aust J Age.

[2] Kosky R (1987). Is suicidal behaviour increasing among Australian youth?. Med J Aust.

[3] McKeown RE, Cuffe SP, Schulz RM (2006). US suicide rates by age group, 1970–2002: an examination of recent trends. Am J Public Health.

[4] McNamara, PM. Adolescent suicide in Australia: rates, risk and resilience. Clin Child Psychol P. 2013;18(3):351–369. 10.1177/1359104512455812 23118313

[5] Beautrais AL (2000). Methods of youth suicide in New Zealand: trends and implications for prevention. Aust NZ J Psychiat.

[6] Shaffer D, Fisher P (1981). The epidemiology of suicide in children and young adolescents. J Am Acad Child Psychiat.

[7] Byard RW, Markopoulos D, Prasad D (2000). Early adolescent suicide: a comparative study. J clin forensic med.

[8] Beautrais AL (2001). Child and young adolescent suicide in New Zealand. Aust NZ J Psychiat.

[9] King RA, Apter A (Eds.). Suicide in children and adolescents. Cambridge University Press; 2003.

[10] Kinoshita K, Kinoshita Y, Shimodera S (2012). Not only body weight perception but also body mass index is relevant to suicidal ideation and self-harming behavior in Japanese adolescents. J Nerve Ment Dis.

[11] Sutin AR, Robinson E, Daly M (2018). Perceived body discrimination and intentional self-harm and suicidal behaviour in adolescence. Child Obes.

[12] United States Census Bureau. American fact finder. ACS demographic and housing estimates. https://factfinder.census.gov/faces/tableservices/jsf/pages/productview.xhtml?pid=ACS_17_5YR_DP05&src=pt

[13] NZ Stat. Subnational population estimates (RC, SA2), by age and sex, at 30 June 1996, 2001, 2006–13, 2018–19 (2019 boundaries). http://nzdotstat.stats.govt.nz/wbos/Index.aspx?DataSetCode=TABLECODE7501&_ga=2.128762316.1326090266.1583743282-604270525.1582535076&_gac=1.224497128.1582539152.CjwKCAiAhc7yBRAdEiwAplGxX4RPzI8VF6u50UW7zbjkCRXT6Rd9ZmH0etFt1g00mMvaHfim_Tj_IxoCVNwQAvD_BwE#

[14] Australian Bureau of Statistics. 2016 Census QuickStats: Greater Adelaide. https://quickstats.censusdata.abs.gov.au/census_services/getproduct/census/2016/quickstat/4GADE?opendocument

[15] Minnesota Department of Health. 2017 Minnesota county health tables. https://www.health.state.mn.us/data/mchs/genstats/countytables/county2017_r2.pdf

[16] Federal Reserve Bank of St Louis Economic Data. Gross domestic product: all industries in Hennepin County, MN. https://fred.stlouisfed.org/series/GDPALL27053

[17] Mishara BL, De Leo D, Schmidtke A, Diekstra RF (1998). Childhood concseptions of death and suicide: empirical investigations and implications of suicide prevention. Suicide prevention: a holistic approach.

[18] Vieweg WV, Linker JA, Anum EA (2005). Child and adolescent suicides in Virginia: 1987 to 2003. J Child Adolesc Psychopharmacol.

[19] Mendes R, Santos S, Taveira F, Dinis-Oliveira RJ, Santos A, Magalhaes T (2015). Child suicide in the north of Portugal. J For Sci.

[20] Canetto S, Sakinofsky I (1998). The gender paradox. Suicide and Life Threatening Behaviour.

[21] Whetstone LM, Morrissey SL, Cummings DM (2007). Children at risk: the association between perceived weight status and suicidal thoughts and attempts in middle school youth. J Sch Health.

[22] Miller M, Azrael D. Access to Firearms Increase Child and Adolescent Suicide. 2020. https://www.srcd.org/research/access-firearms-increases-child-and-adolescent-suicide 10.1136/bmj.m2829 32699044

[23] Zainum K, Cohen MC (2017). Suicide patterns in children and adolescents: a review from a pediatric institution in England. Forensic Sci Med Pathol.

[24] Byard RW, Haas E, Marshall DT, Gilbert JD, Krous HF (2009). Characteristic features of pediatric firearm fatalities – comparisons between Australia and the United States. J Forensic Sci.

